# Pancreas deficiency modifies bone development in the ovine fetus near term

**DOI:** 10.1530/JOE-21-0189

**Published:** 2021-10-27

**Authors:** Stuart A Lanham, Dominique Blache, Richard O C Oreffo, Abigail L Fowden, Alison J Forhead

**Affiliations:** 1 Bone and Joint Research Group, Centre for Human Development, Stem Cells and Regeneration, School of Medicine, University of Southampton, Southampton, UK; 2 School of Agriculture and Environment, University of Western Australia, Crawley, Australia; 3 Department of Physiology, Development and Neuroscience, University of Cambridge, Cambridge, UK; 4 Department of Biological and Medical Sciences, Oxford Brookes University, Oxford, UK

**Keywords:** fetus, pancreas, insulin, glucose, leptin, micro-computed tomography, bone structure, metatarsal, skeleton

## Abstract

Hormones have an important role in the regulation of fetal growth and development, especially in response to nutrient availability *in utero*. Using micro-CT and an electromagnetic three-point bend test, this study examined the effect of pancreas removal at 0.8 fraction of gestation on the developing bone structure and mechanical strength in fetal sheep. When fetuses were studied at 10 and 25 days after surgery, pancreatectomy caused hypoinsulinaemia, hyperglycaemia and growth retardation which was associated with low plasma concentrations of leptin and a marker of osteoclast activity and collagen degradation. In pancreatectomized fetuses compared to control fetuses, limb lengths were shorter, and trabecular (Tb) bone in the metatarsi showed greater bone volume fraction, Tb thickness, degree of anisotropy and porosity, and lower fractional bone surface area and Tb spacing. Mechanical strength testing showed that pancreas deficiency was associated with increased stiffness and a greater maximal weight load at fracture in a subset of fetuses studied near term. Overall, pancreas deficiency *in utero* slowed the growth of the fetal skeleton and adapted the developing bone to generate a more compact and connected structure. Maintenance of bone strength in growth-retarded limbs is especially important in a precocial species in preparation for skeletal loading and locomotion at birth.

## Introduction

Growth and development of long bones during late gestation are important for the normal functioning of the neonatal skeleton, especially in precocial species like sheep that stand and are mobile from birth. In normal and adverse intrauterine conditions, a number of hormones are known to regulate skeletal growth and development *in utero* to optimize the size, structure and mechanical strength of bone (Agrogiannis *et al.* 2014, Kovacs 2014). The majority of studies have focused, however, on the endocrine control of bone development in altricial species where the skeleton is not required for weight-bearing or locomotion until later in postnatal life.

Insulin is a key growth hormone in the fetus. It is secreted by the fetal pancreas in response to changes in nutrient availability and coordinates appropriate growth and development of fetal tissues, including the skeleton (Fowden 1992, Sferruzzi-Perri *et al.* 2013). A range of experimental animal models have demonstrated that suboptimal intrauterine conditions, such as undernutrition and/or hypoxia, are associated with impaired insulin secretion and skeletal growth retardation and have investigated further the role of insulin in the control of fetal growth in large animals (Fowden 1993, Boehmer *et al.* 2017). In fetal sheep, surgical removal of the pancreas or treatment with streptozocin to damage pancreatic β-cells causes growth retardation of the axial and appendicular skeleton, delayed ossification of limb bones as assessed by radiography, proportionate growth restriction of organs and sparing of the growth of the brain (Brinsmead & Thorburn 1982, Fowden *et al.* 1989, Philipps *et al.* 1991). In these studies, the percentage reduction in body weight was greater than the percentage reduction in body and limb lengths. The findings suggest that there are tissue-specific effects, and relative preservation of skeletal growth, following insulin deficiency in fetal sheep.

In postnatal life, insulin contributes to the regulation of bone formation and remodelling (Pramojanee *et al.* 2014), although relatively less is known about its actions on the skeleton before birth, especially in precocial offspring. Studies *in vitro* have demonstrated that insulin stimulates collagen and DNA synthesis and bone matrix formation in rat fetuses near term and proliferation of chondrocytes isolated from ovine fetal growth plates (Hock *et al.* 1988, Hill & de Sousa 1990). To date, however, the role of insulin in determining the structural and mechanical properties of developing bone near term has not been investigated in detail in large animals.

This study aims to investigate the effects of hypoinsulinaemia *in utero*, induced by removal of the fetal pancreas, on aspects of the structure and mechanical strength of bone in the sheep fetus using micro-CT and an electromagnetic three-point bend test. It tests the hypothesis that pancreas deficiency will impair the structural organization and development of fetal bone with consequences for the growth of the fetal skeleton and its weight-bearing capacity. Pancreas deficient and control fetuses were examined at two ages during late gestation, at the start and during the plateau phase of skeletal growth observed shortly before birth in sheep (Fowden *et al.* 1996).

## Methods

### Animals

This study used the sheep as an example of a precocial species where the skeleton is weight-loaded and required for motility at birth and where circulating insulin concentrations in the fetus can be manipulated experimentally over a period of late gestation by pancreatectomy. This large animal model of insulin deficiency *in utero* has been characterized previously, in terms of aspects of fetal growth and metabolism (Fowden 1993, Fowden & Forhead 2012).

A total of 24 Welsh Mountain sheep fetuses of known gestational age were used in the study. There were 13 singleton and 11 twin fetuses distributed evenly across 4 groups (sham and pancreatectomized (PX), at 2 gestational ages: 129 and 144 days of gestation, where term is 145 ± 2 days; Table 1). In four ewes with a twin pregnancy, the pancreas was removed in one fetus while the other acted as the sham-operated control; in three twin-bearing ewes, only one of the fetuses was included in the study, in either sham or PX groups. No siblings were included in the same treatment group. The pregnant ewes were housed individually and maintained on 200 g/day concentrates with free access to hay, water and a salt-lick block. Food, but not water, was withheld for 18–24 h before surgery. All surgical and experimental procedures were carried out in accordance with the UK Animals (Scientific Procedures) Act 1986 and approved by the animal ethics committee at the University of Cambridge.

**Table 1:** Mean (± s.e.m.) measurements of bodyweight, crown-rump and limb lengths, and plasma hormone and metabolite concentrations, in sham and PX sheep fetuses at 129 and 144 days of gestation.

Gestational age (days)	129		144		Two-way ANOVA analyses		
Treatment	Sham	PX	Sham	PX	Treatment	Gestational age	Interaction
Number of fetuses	8 (4F:4M; 4T:4S)	6 (2F:4M; 3T:3S)	5 (1F:4M; 2T:3S)	5 (2F:3M; 2T:3S)			
Body weight (kg)	2.71 ± 0.18	2.41 ± 0.11	3.74 ± 0.25^a^	3.17 ± 0.23^a^	*P* < 0.05	*P* < 0.001	NS
Crown-rump length (cm)	42.6 ± 1.2	42.2 ± 1.1	50.8 ± 1.7^a^	47.2 ± 1.8^a^	NS	*P* < 0.001	NS
							
Total hind-limb length (cm)	40.4 ± 1.1	38.8 ± 1.0	46.8 ± 1.5^a^	41.4 ± 1.3^b^	*P* < 0.01	*P* < 0.001	NS
Metatarsus/phalanges (cm)	15.6 ± 0.4	14.8 ± 0.3	17.8 ± 0.8^a^	16.0 ± 0.6^b^	*P* < 0.01	*P* < 0.001	NS
Tibia (cm)	13.4 ± 0.4	13.0 ± 0.3	15.8 ± 0.4^a^	13.6 ± 0.4^b^	*P* < 0.005	*P* < 0.001	*P* < 0.05
Femur (cm)	11.4 ± 0.4	11.0 ± 0.5	13.2 ± 0.7^a^	11.8 ± 0.4	NS	*P* < 0.05	NS
Total fore-limb length (cm)	33.1 ± 0.8	31.3 ± 1.0	36.4 ± 1.0^a^	34.4 ± 1.5	NS	*P* < 0.01	NS
Metacarpus/phalanges (cm)	13.2 ± 0.3	12.2 ± 0.3^b^	14.4 ± 0.2^a^	13.2 ± 0.6^b^	*P* < 0.01	*P* < 0.005	NS
Radius (cm)	10.8 ± 0.3	10.3 ± 0.4	12.0 ± 0.3^a^	11.2 ± 0.5	NS	*P* < 0.05	NS
Humerus (cm)	9.1 ± 0.3	8.8 ± 0.4	10.0 ± 0.5	10.0 ± 0.5	NS	*P* < 0.05	NS
							
Insulin (ng/mL)	0.30 ± 0.06	0.05 ± 0.01^b^	0.34 ± 0.17	0.04 ± 0.01^b^	*P* < 0.005	NS	NS
Glucose (mmol/L)	0.71 ± 0.08	1.02 ± 0.09	0.73 ± 0.13	1.49 ± 0.32^b^	*P* < 0.005	NS	NS
Cortisol (ng/mL)	11.6 ± 1.8	12.2 ± 2.1	76.3 ± 17.2^a^	36.2 ± 7.1^a^	NS	*P* < 0.001	NS
T4 (ng/mL)	87.7 ± 10.9	74.3 ± 8.8	65.5 ± 8.7	43.4 ± 4.6^a^	NS	*P* < 0.05	NS
T3 (ng/mL)	0.18 ± 0.02	0.16 ± 0.02	0.55 ± 0.16^a^	0.30 ± 0.03^a^	NS	*P* < 0.001	NS
Leptin (ng/mL)	0.90 ± 0.08	0.76 ± 0.04	1.06 ± 0.07	0.71 ± 0.04^b^	*P* < 0.005	NS	NS
CTX (ng/mL)	0.76 ± 0.04	0.68 ± 0.07	0.99 ± 0.24	0.43 ± 0.06^b^	*P* < 0.05	NS	*P* < 0.05
Osteocalcin (ng/mL)	12.2 ± 1.2	12.9 ± 0.3	11.9 ± 1.5	13.1 ± 0.8	NS	NS	NS
Calcium (mmol/L)	2.34 ± 0.28	2.97 ± 0.15	2.73 ± 0.32	3.38 ± 0.28	*P* = 0.053	NS	NS

^a^Significant difference from fetuses of the same treatment at 129 days of gestation, two-way ANOVA (*P* < 0.05); ^b^Significant difference from sham fetuses at the same gestational age (*P* < 0.05). F, female; M, male; NS, not significant; S, singleton; T, twin.

### Surgical procedures

The pregnant ewes were fasted, with access to water, for 18–24 h before surgery. Surgical operations were carried out under general anaesthesia (1.5% halothane in O_2_–N_2_O) with positive pressure ventilation. Between 115 and 120 days of gestation, fetuses were either pancreatectomized (PX, *n*  = 11) or sham-operated (sham, *n*  = 13), where the pancreas was exposed but not removed (Fowden & Comline 1984). Catheters were inserted into the fetal aorta and vena cava and the maternal aorta, via the femoral vessels and exteriorized through the flank of the ewe and secured in a bag sutured to the skin, as described previously (Comline & Silver 1972).

At surgery, all fetuses were administered 100 mg ampicillin i.v. (Penbritin; Beecham Animal Health, Brentford, UK) and 2 mg gentamicin i.v. (Frangen-100; Biovet, Mullingar, Ireland). The ewes were given antibiotics i.m. (procaine penicillin, Depocillin; Mycofarm, Cambridge, UK) on the day of surgery and daily for the next 3 days. All vascular catheters were flushed daily with heparinized saline solution (100 IU heparin in 0.9% (w/v) saline) from the day after surgery. During the study, daily blood samples collected from these catheters were used to monitor fetal wellbeing by measuring blood gases and pH.

### Tissue collection

The PX and sham fetuses were delivered by Caesarean section under general anaesthesia (20 mg/kg sodium pentobarbitone i.v. to the ewe) at either 127–131 days (129 days; PX *n*  = 6, sham *n*  = 8) or 142–145 days of gestation (144 days; PX *n*  = 5, sham *n*  = 5). At delivery, 5 mL of blood samples were taken by venepuncture of the umbilical artery and placed into EDTA-containing tubes. The samples were centrifuged for 5 min at 1000 ***g*** at 4°C, and the plasma aliquots were stored at −20°C until analysis.

The fetuses were administered with a lethal dose of barbiturate (200 mg/kg sodium pentobarbitone), and morphometric measurements were made at dissection during post-mortem. Crown-rump length (CRL) and lengths of the hind-limb (femur, tibia and metatarsus/phalanges) and fore-limb (humerus, radius and metacarpus/phalanges) were measured. The metatarsal bone from one hind-limb of each fetus was immediately frozen in liquid nitrogen and stored at −80°C until analysis. In the sheep, each hind-limb has one elongated metatarsal bone formed from the fusion of metatarsi III and IV. Metatarsi I and V are absent, and metatarsus II is a small vestigial bone. At delivery, there was no evidence of pancreatic remnants in any of the PX fetuses.

### Biochemical analyses

Umbilical plasma insulin concentration was determined using an ELISA kit (Mercodia, Uppsala, Sweden); the intra-assay coefficient of variation was 9%, and the minimum level of detection was 0.025 ng/mL. Plasma cortisol and leptin concentrations were measured by RIA as previously described (Robinson *et al.* 1983, Blache *et al.* 2000). The intra-assay coefficients of variation were 11 and 5%, and the minimum levels of detection were 1.5 and 0.09 ng/mL, respectively. Plasma triiodothyronine (T3) and thyroxine (T4) concentrations were determined by RIA kits (MP Biomedicals, Loughborough, UK); the intra-assay coefficients of variation were 3 and 5%, and the minimum levels of detection were 0.14 and 7.0 ng/mL, respectively. Plasma glucose concentration was measured using a Yellow Springs glucose analyser (2300 Statplus, YSI Incorporated, Yellow Springs, Ohio, USA).

Plasma levels of total osteocalcin and the degradation products of C-terminal telopeptides of type I collagen (CTX) were determined by ELISA (Immunodiagnostics Systems Ltd, Boldon, UK). The lower limits of detection of osteocalcin and CTX were 0.5 and 0.02 ng/mL, respectively, and all measurements were made in a single assay. Total plasma calcium was measured by a Siemens Dimension RXL auto-analyser using Siemens reagents and calibrators (Siemens Healthcare, Camberley, UK).

### Micro-computed tomography

The fetal metatarsi were scanned using an Xtek Benchtop 160Xi scanner (Xtek Systems Ltd, Tring, UK) equipped with a Hamamatsu C7943 X-ray flat panel sensor (Hamamatsu Photonics, Welwyn Garden City, UK) as described previously (Lanham *et al.* 2011*b*). Reconstructed images were analysed using VGStudio Max 1.2.1 software (Volume Graphics GmbH, Heidelberg, Germany) to calculate the bone volume to total volume ratio (BV/TV), bone surface area to bone volume ratio (BS/BV), trabecular (Tb) thickness, spacing and number per unit length. Using a custom written package and the Visilog Quantification + package (both Noesis, Crolles, France) within the Amira 4.1.2 package (Mercury Computer System Inc., Chelmsford, USA), measurements were also made of porosity, Euler number (a measure of connectivity), structural model index (a measure of surface convexity where an ideal plate, cylinder and sphere have SMI values of 0, 3 and 4, respectively), Tb pattern factor (the relative concave or convex nature of the total bone surface, where concavity indicates connectivity and convexity indicates isolated, disconnected structures), average object (Av Obj) number and area (indicators of structural connectivity, where high connectivity results in few and large objects, while fragmentation results in large numbers of smaller), degree of anisotropy (DOA, orientation of trabeculae) and fractal dimension.

### Bone strength testing

Mechanical bone strength was assessed in a subset of fetuses at 144 days of gestation (*n* = 4 from each treatment group). The midshaft strength of metatarsal cortical bone was tested using an electromagnetic bend test instrument (Bose Electroforce 3200, Bose Corporation, Minnesota, USA). The bones were placed with anterior surface down on two supports, 40 mm apart and equidistant from the bone ends. Each bone was loaded centrally at a constant rate (6 mm/min) until fracture. In order to test the strength of Tb bone, a small block of Tb bone (3 × 3m × 6 mm^3^) was cut from the distal end of the metatarsal. The sample was placed between two supports and loaded at a constant rate (1 mm/min) until failure. Load–displacement curves were used to calculate maximum load at fracture, maximum displacement at fracture, stiffness and stress. Stiffness was determined as the slope of the linear portion of the load–displacement curve, while stress was calculated as the maximum load divided by the cross-sectional area as measured by micro-CT.

### Statistical analysis

Data are presented as mean ± s.e.m. and were assessed for normality using the D’Agostino–Pearson’s test. Log_10_-transformed data were analysed where necessary. The effect of PX at 129 and 144 days of gestation was assessed by two-way ANOVA with treatment and gestational age as factors, followed by the Tukey’s test. Bone strength properties were compared between treatment groups at 144 days of gestation by Student’s *t*-test. Relationships between variables were determined by Spearman correlation. Plasma hormone concentrations below the minimum level of detection were assigned the minimum value for the presentation and statistical analysis of the data. Statistical significance was accepted at *P* < 0.05.

## Results

### Plasma hormone and metabolite concentrations

Umbilical plasma insulin concentration in the PX fetuses was near to the minimum limit of assay detection and was lower than that observed in the sham control fetuses at both gestational ages (log_10_ insulin, Table 1, *P* < 0.05). Plasma concentrations of leptin and CTX were lower, and glucose was higher, in the PX compared to sham fetuses at 144 days of gestation (Table 1, *P* < 0.05). Towards term, a decrease in plasma T4 in the PX fetuses (*P* < 0.05) and increments in plasma concentrations of cortisol and T3 in both sham and PX fetuses (log_10_ cortisol and T3, *P* < 0.05) were observed with no differences in these concentrations between the treatment groups at either gestational age (Table 1). There were no significant effects of gestational age or treatment on plasma calcium or osteocalcin concentrations, although there was a tendency for higher plasma calcium concentration in the PX compared to sham fetuses which did not reach significance (log_10_ calcium, *P* = 0.053).

When all data were combined, regardless of treatment or gestational age (*n* = 24 fetuses), significant relationships were observed between plasma insulin and plasma concentrations of glucose (R = −0.59, *P* < 0.005), leptin (R = +0.57, *P* < 0.005) and CTX (R = +0.42, *P* < 0.05). Plasma leptin was negatively correlated with osteocalcin (R = −0.45, *P* < 0.05). Overall, plasma cortisol concentration correlated positively with plasma T3 (R = +0.62, *P* < 0.005) and negatively with plasma T4 (R = −0.52, *P* < 0.01). Positive correlations were observed between plasma T3 and calcium concentrations (R = +0.44, *P* < 0.05) and between plasma T4 and CTX concentrations (R = +0.44, *P* < 0.05).

### Fetal morphology

Fetal body weight and CRL increased towards term in both treatment groups. Overall, body weight was reduced in the PX compared to sham fetuses, although the *post hoc* analysis failed to identify significant differences with treatment at either gestational age (Table 1, *P* = 0.067 at 144 days). Total and individual bone lengths of the fore- and hind-limbs increased with gestational age in the sham, but not PX fetuses (Table 1, *P* < 0.05). At 144 days of gestation, measurements of the total hind-limb, metatarsus-phalanges, tibia and metacarpus-phalanges were lower in the PX compared to sham fetuses (Table 1, *P* < 0.05); the length of the metacarpus-phalanges was also reduced in the PX fetuses studied at 129 days of gestation (Table 1, *P* < 0.05).

### Metatarsal bone structure

**Figure 1:**
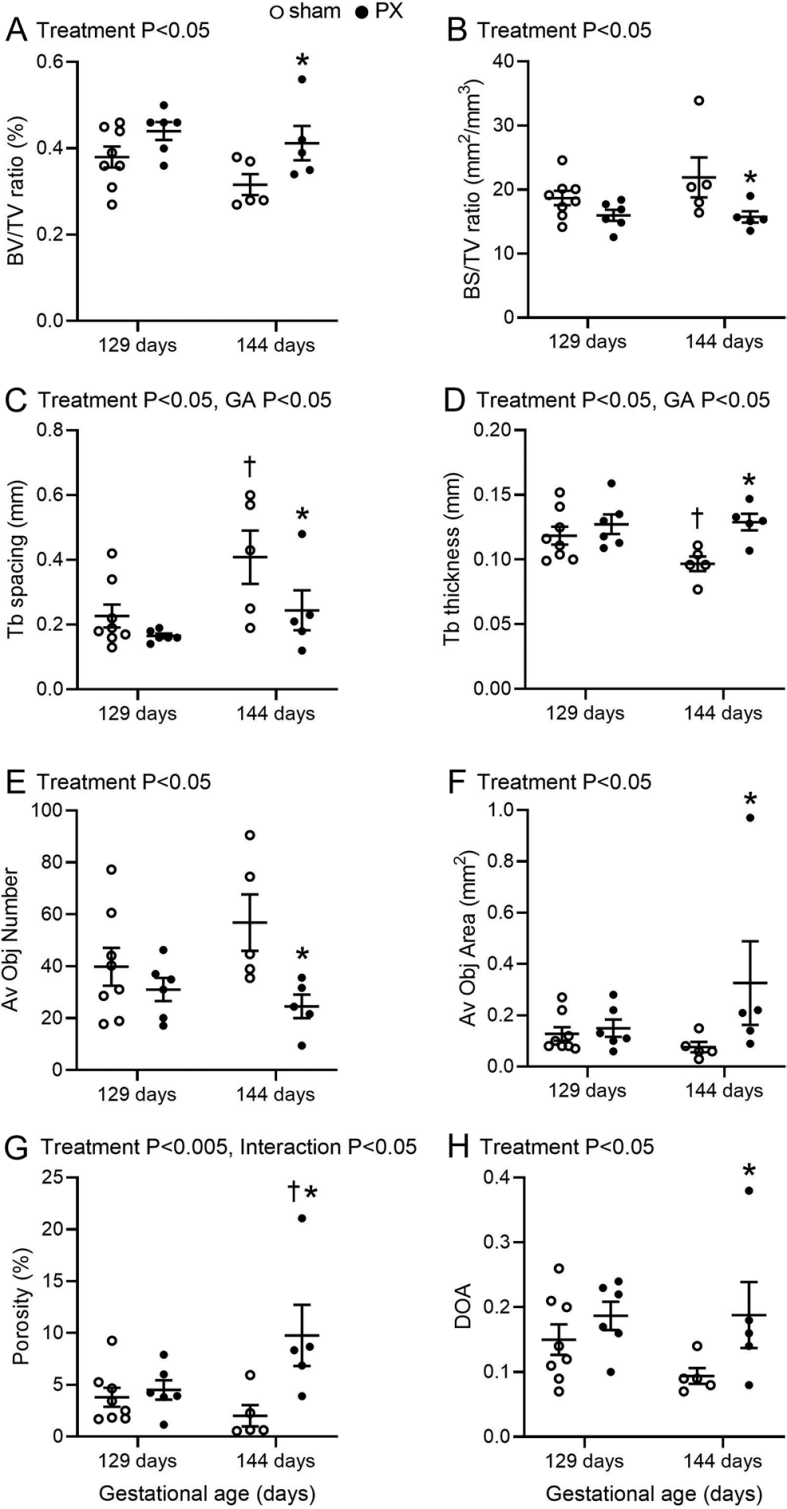
Mean (± s.e.m.) BV/TV (A), ratio of BS/BV (B), Tb spacing (C), Tb thickness (D), Av Obj number (E), average object area (F), porosity (G) and DOA (H) in the metatarsal bone of sham and PX fetuses at 129 and 144 days of gestation. *Significant difference from sham fetuses at the same gestational age (*P* < 0.05). †Significant difference from fetuses of the same treatment at 129 days of gestation (*P* < 0.05). BV/TV, bone volume–total volume ratio; BS/BV, bone surface area–bone volume ratio; Tb, trabecular; Av Obj, average object; DOA, degree of anisotropy.

**Figure 2:**
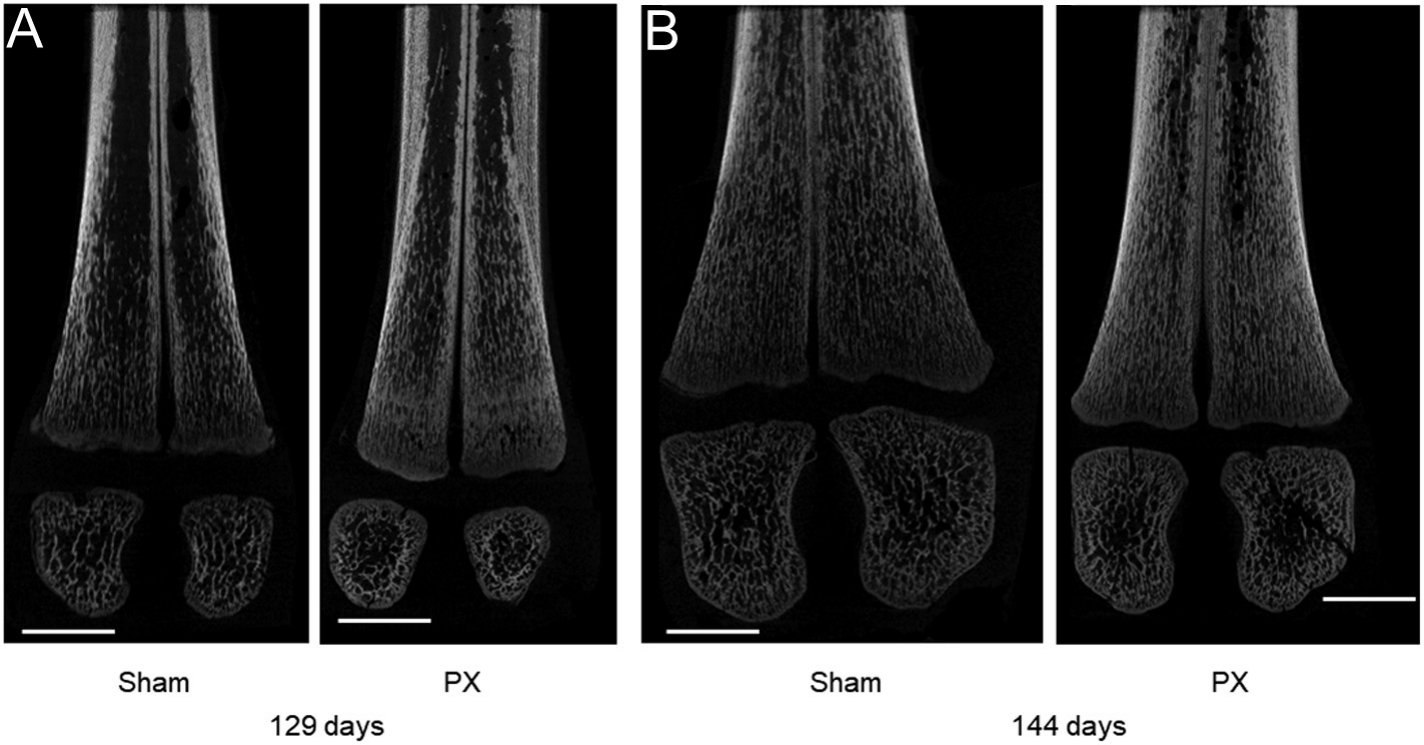
Representative images of longitudinal sections of the distal metatarsal bone showing trabecular structure and secondary ossification sites in sham and PX fetuses at (A) 129 and (B) 144 days of gestation. Bars represent 5 mm.

Fetal pancreatectomy had significant effects on the aspects of bone structure, including BV/TV and BS/BV ratios, Tb spacing and thickness, Av Obj number and area, DOA and porosity (Fig. 1, *P* < 0.05). In the PX fetuses, BV/TV ratio, Tb thickness, Av Obj area, DOA and porosity were higher, and BS/BV ratio, Tb spacing and Av Obj number were lower, compared to the sham fetuses at 144 days of gestation (Fig. 1, *P* < 0.05). Between 129 and 144 days of gestation, Tb spacing increased in the sham fetuses, and porosity increased in the PX fetuses (Table 2, *P* < 0.05). There were no effects of treatment or gestational age on the measurements of Euler, SMI, Tb number, Tb pattern factor or fractal dimension (Table 2). Representative images of longitudinal sections of metatarsal bones in PX and sham fetuses at 129 and 144 days of gestation are presented in Fig. 2.

**Table 2:** Mean (± s.e.m.) measurements of metatarsal trabecular bone structure in sham and PX sheep fetuses at 129 and 144 days of gestation. No significant differences identified by two-way ANOVA (*P* > 0.05).

Gestational age (days)	129		144	
Treatment	Sham	PX	Sham	PX
Number of fetuses	8	6	5	5
Trabecular number (mm)	3.23 ± 0.18	3.46 ± 0.12	3.24 ± 0.17	3.19 ± 0.18
Trabecular pattern factor	−4.14 ± 0.51	−4.33 ± 0.75	−2.37 ± 0.79	−4.79 ± 1.76
Structural model index	0.15 ± 0.02	0.14 ± 0.02	0.09 ± 0.02	0.16 ± 0.04
Euler	−1897 ± 432	−1113 ± 268	−1360 ± 301	−985 ± 159
Fractal dimension	2.51 ± 0.04	2.51 ± 0.03	2.37 ± 0.04	2.50 ± 0.05

Overall, relationships were observed between plasma insulin and BS/BV ratio (R = +0.50, *P* < 0.05), Av Obj number (R = +0.47, *P* < 0.05), porosity (R = −0.44, *P* < 0.05) and Euler (R = −0.51, *P* < 0.05) and between plasma leptin and BV/TV ratio (R = −0.55, *P* < 0.01), BS/BV ratio (R = +0.61, *P* < 0.005), Av Obj number (R = +0.51, *P* < 0.05) and area (R = −0.54, *P* < 0.01), Tb spacing (R = +0.47, *P* < 0.05) and thickness (R = −0.67, *P* < 0.0005), porosity (R = −0.52, *P* < 0.01), DOA (R = −0.50, *P* < 0.05) and fractal dimension (R = −0.49, *P* < 0.05).

### Metatarsal bone strength

Table 3 shows that, compared to the sham fetuses, there were no significant differences in the maximum load, maximum displacement, stiffness or stress in the midshaft cortical bone between the sham and PX fetuses at 144 days of gestation. The distal Tb bone in the PX fetuses fractured at a greater maximum load, per unit area, compared to that in the sham fetuses (*P* < 0.05; Table 3). Furthermore, Tb bone in the PX fetuses showed higher stiffness than in the sham fetuses (*P* < 0.05; Table 3). Maximum displacement and stress in Tb bone did not differ between sham and PX fetuses (Table 3).

**Table 3:** Mean (± s.e.m.) measurements of bone strength in metatarsal midshaft cortical and distal trabecular bone from sham and PX fetuses at 144 days of gestation.

Treatment	Sham	PX
Number of fetuses	4	4
Midshaft cortical bone		
Maximum load (N)	135.8 ± 11.7	132.3 ± 8.2
Maximum displacement (mm)	2.99 ± 0.37	3.46 ± 0.21
Stiffness (N/mm)	81.0 ± 17.3	55.2 ± 13.0
Stress (N/mm^2^)	2.04 ± 0.10	2.15 ± 0.24
Distal trabecular bone		
Maximum load (N)	21.0 ± 6.2	56.2 ± 11.7^a^
Maximum displacement (mm)	0.69 ± 0.11	0.84 ± 0.03
Stiffness (N/mm)	41.4 ± 3.1	90.1 ± 1.2^a^
Stress (N/mm^2^)	282.8 ± 54.2	489.0 ± 132.9

^a^Significant difference, Student’s *t*-test (*P* < 0.05).

When all the data were combined (*n* = 8 fetuses), significant correlations were observed between plasma T4 and both maximum displacement (R = −0.76, *P* < 0.05) and stiffness (R = +0.74, *P* < 0.05) in midshaft cortical bone. Stress in cortical bone was correlated with plasma concentrations of calcium (R = +0.96, *P* < 0.01), CTX (R = −0.83, *P* < 0.05) and osteocalcin (R = +0.81, *P* < 0.05). In distal Tb bone, significant relationships were observed between plasma T3 and both maximal load (R = −0.86, *P* < 0.05) and stress (R = −0.93, *P* < 0.01).

## Discussion

Pancreas deficiency in the sheep fetus over the last 20–35 days of gestation reduced body weight and growth of the appendicular skeleton. In the hind-limb, the shorter metatarsal bone length was associated with changes to the microarchitecture of the Tb bone. The increase in Tb BV/TV ratio indicated a more compact bone structure with a relatively low internal bone surface area (BS/BV ratio), likely due to an increase in Tb thickness. Although the trabeculae were larger, they were more closely spaced together within the bone of the pancreas-deficient fetus, with enhanced porosity evident near term. The increase in DOA also indicated that the spatial organization of the trabeculae was predominantly uni-directional, rather than multi-directional, in orientation. Many of the effects of PX were significant at 144 days, and not 129 days, of gestation which may reflect the duration of pancreas deficiency. In a subset of fetuses studied near term, mechanical testing showed that distal Tb bone samples were more stiff and fractured at a greater maximal load in the PX compared with sham fetuses. Overall, these indices demonstrated that, while the bone size was reduced in response to pancreas deficiency *in utero*, metatarsal Tb bone structure was more condensed with greater structural connectivity which may be a key adaptation to maintain bone strength in precocial offspring. Use of a large animal model and measurement of a number of structural, biochemical and mechanical end-points are key strengths of this study which enabled the assessment of the consequences of hypoinsulinaemia, secondary to pancreas deficiency, in a precocial species over a period of late gestation. As a limitation, the relatively low number of fetuses in each treatment group may have weakened the statistical analysis and accounted for tendencies in plasma calcium and glucose concentrations.

In this study, the role of insulin in the control of bone growth and development in the fetus near term was examined by removal of the fetal pancreas which caused hypoinsulinaemia. Previous studies in fetal sheep have shown that pancreas deficiency reduces fore- and hind-limb lengths, and the daily increment in CRL measured using indwelling growth catheters, and that axial and appendicular growth can be restored to normal rates by insulin replacement (Fowden *et al.* 1989). In the present study, however, there were no effects of pancreatectomy on CRL, although this was a single measurement taken in a relatively small number of fetuses at delivery and the microstructure of the vertebrae was not investigated. Normalization of growth by insulin replacement in the PX sheep fetus suggests that the primary deficiency of PX in relation to bone growth appears to be hypoinsulinaemia. However, specific skeletal effects may be mediated via other insulin-dependent factors. Indeed, insulin replacement is also likely to normalize other endocrine and metabolite changes in the PX fetus that may contribute to the effects of pancreas deficiency on bone growth and development.

In adult life, insulin influences skeletal remodelling via actions on the proliferation, differentiation and activity of both bone and cartilage cell types (Pramojanee *et al.* 2014). Much less is known, however, about the targets for insulin action before birth, especially in precocial, large animals. In the present study, the plasma concentration of osteocalcin, a marker of osteoblast activity and bone formation, was unchanged by pancreas deficiency while CTX, a marker of osteoclast activity and bone resorption, was lower in the PX fetuses. Plasma CTX increases in intact sheep fetuses over the last month of gestation (Lanham *et al.* 2011*b*) and this developmental rise appeared to be prevented in the PX fetus. Circulating concentrations of both CTX and osteocalcin are reduced in mice with targeted deletion of the insulin receptor in osteoblasts (Ferron *et al.* 2010, Fulzele *et al.* 2010). At 3–12 weeks of postnatal age, BV/TV ratio was reduced in the femur of these mutant mice with fewer, thinner trabeculae and reductions in osteoblast number and resorption sites (Ferron *et al.* 2010, Fulzele *et al.* 2010). Further research is required to elucidate the cellular and molecular mechanisms responsible for changes in bone development associated with pancreas, and specifically insulin, deficiency *in utero* in both precocial and altricial species.

In addition to the direct effects of hypoinsulinaemia, there are a number of metabolic and other endocrine factors that may be responsible for the changes in bone growth and development due to intrauterine pancreas deficiency. In fetal sheep, hypoinsulinaemia induced either by pancreas removal or streptozocin treatment is associated with hyperglycaemia and decreases in umbilical glucose uptake (both absolute and relative to fetal body weight), fetal glucose utilization and the fraction of umbilical oxygen uptake used to oxidize glucose carbon, all of which can be restored to normal levels by insulin replacement (Fowden & Hay 1988, Hay *et al.* 1989, Fowden & Forhead 2012). In these models of insulin deficiency, the reduction in glucose utilization leads to greater use of other substrates for metabolism, including amino acids. This means that there are less amino acids available for protein synthesis in bone and other tissues. Furthermore, the increase in circulating glucose concentration that accompanies insulin deficiency may contribute to the changes in bone structure observed in the PX fetuses as more severe hyperglycaemia in diabetic models and studies *in vitro* is associated with abnormalities in bone development (Dienelt & zur Nieden 2011, Seref-Ferlengez *et al.* 2016). It is difficult, however, to establish the relative roles of hypoinsulinaemia and hyperglycaemia in diabetic models *in vivo*, and variable results have been reported from a range of experimental approaches *in vitro* (reviewed by Seref-Ferlengez *et al.* 2016). In a mouse model where the insulin receptor was deleted but blood glucose concentration was normalized by the expression of human insulin receptor transgene in the pancreas, liver and brain, bone development was relatively normal at 4–5 months of postnatal age (Irwin *et al.* 2006). Although the insulin receptor was not present in bone, there were no differences in cortical or Tb structure as assessed by micro-CT. Overall, the findings suggest that hyperglycaemia may be responsible, at least in part, for the abnormal skeletal growth associated with impaired insulin signalling in adulthood. It was also noted, however, that expression of the insulin-like growth factor type I (IGFI) receptor was upregulated in the bone of mutant mice which may have driven skeletal development in response to IGFs and/or the high circulating concentrations of insulin seen in this model (Irwin *et al.* 2006). There are, therefore, a variety of other factors that affect bone structure which need to be considered in models of hypoinsulinaemia, including targeted gene mutations.

The IGFs may contribute to the changes in bone structure observed in the PX fetus (Agrogiannis *et al.* 2014, Pramojanee *et al.* 2014, Yakar *et al.* 2018). Although not measured in the present study, it has been shown previously that plasma concentrations of IGFI are decreased and insulin-like growth factor II (IGFII) are increased in the PX sheep fetus, with positive and negative correlations observed between plasma insulin and concentrations of IGFI and IGFII, respectively (Gluckman *et al.* 1987). Similarly, in a clinical case of transient neonatal diabetes mellitus where the infant was born with IUGR and umbilical plasma concentrations of insulin were undetectable, IGFI was lower than normal and IGFII was within the normal range (Blethen *et al.* 1981). In fetal sheep, IGFs and their binding proteins are co-localized in the epiphyseal growth plates, and IGFI and IGFII have been shown *in vitro* to stimulate the proliferation of chondrocytes isolated from the growth plates (Hill & de Sousa 1990, de los Rios & Hill 1999). In this model system, insulin also increases the expression of IGF-binding protein-3 which may modify the bioavailability and paracrine actions of IGFs within the developing bone (Rios & Hill 2000). IGFI has been shown to increase collagen and DNA synthesis and bone matrix formation *in vitro* in skull calvariae taken from rat fetuses close to term (Hock *et al.* 1988). *In vivo* infusion of IGFI in sheep fetuses promotes epiphyseal development, although there were no overall effects on limb length or weight, and plasma insulin and IGFII concentrations were reduced (Lok *et al.* 1996). In fetal mice with deletions in IGFI or the type 1 receptor, IGF-1R, growth retardation was associated with a delay in skeletal ossification, both of which were more marked in the IGF-1R mutants (Liu *et al.* 1993). Analysis of Tb bone structure using micro-CT showed reductions in BV/TV ratio, Tb spacing and DOA in the tibia of IGFI-deficient mouse fetuses (Burghardt *et al.* 2007).

In the present study, plasma leptin concentration was reduced in the PX fetus and correlated with several of the Tb bone indices. Like insulin, leptin has been proposed as a mechanism linking bone remodelling with nutrient availability before and after birth (Forhead & Fowden 2009, Devlin & Bouxsein 2012), and a relationship between circulating insulin and adipose leptin mRNA has been shown previously in fetal sheep (Devaskar *et al.* 2002). Infusion of supraphysiological concentrations of leptin influences metatarsal Tb structure in fetal sheep, although leptin receptor antagonism only affected the development of the vertebra, and not the metatarsal or femur (De Blasio *et al.* 2018). Further studies are required to determine the effects of intrauterine pancreas deficiency on leptin signalling within the developing skeleton and to assess the relative contribution of low circulating leptin on bone structure and function in this model.

The changes in Tb bone structure and mechanical strength observed in the pancreas-deficient ovine fetus resemble those seen in skeletal growth retardation associated with hypothyroidism *in utero* (Lanham *et al.* 2011*b*) and indicate that there may be a common mechanism that leads to modified skeletal growth and development in both models of fetal endocrine deficiency. Similarities in bone development occurred even though the hypothyroid sheep fetus has high circulating concentrations of insulin (Harris *et al.* 2017), and plasma thyroid hormones appear to be unaffected by pancreas deficiency. Local hormone concentrations and the activity of signalling pathways, however, including those of IGF and other growth factors, remain to be determined in the developing Tb bone of both pancreas- and thyroid-deficient sheep fetuses.

Insulin and thyroid hormones are important anabolic signals in the fetus that respond to changes in nutrient levels and promote tissue growth when substrates are available (Sferruzzi-Perri *et al.* 2013). In conditions of undernutrition *in utero*, circulating concentrations of these hormones are reduced (Sferruzzi-Perri *et al.* 2013), and the resultant change in skeletal development, by common or different mechanisms, may favour a more compact bone structure that maintains mechanical integrity in a smaller bone. Indeed, in both sheep models of pancreas and thyroid deficiency *in utero*, mechanical stress tests showed that metatarsal Tb bone was stronger per unit area, albeit more stiff and brittle, than in control fetuses (Lanham *et al.* 2011*b*). During these endocrine conditions, changes in Tb bone microstructure may be an important adaptation that preserves bone strength in the face of limited availability of substrates for bone growth. This is likely to be of particular importance in a precocial species like the sheep where the neonatal skeleton is required for locomotion from immediately after birth. In contrast, bone development and strength may be relatively more compromised by pancreas deficiency in altricial rodents and human offspring that do not require weight-bearing bones at birth. Importantly, the need for skeletal maturity and function at birth may account for some of the differences in fetal bone development identified between mammalian species (Lanham *et al.* 2011*a*).

To conclude, fetal hypoinsulinaemia associated with pancreas deficiency retarded growth of the limbs and adapted the developing bone to a more compact and connected structure that maintained mechanical strength. Further studies are required to establish the molecular mechanisms responsible and whether the bone phenotype established in response to pancreas deficiency *in utero* has longer-term consequences for postnatal bone development, at a time when there are changes to nutrient supply and growth rate, endocrine environment and locomotive activity.

## Declaration of interest

The authors declare that there is no conflict of interest that could be perceived as prejudicing the impartiality of the research reported.

## Funding

This work was supported by the BBSRC (grant number S18103) and Research into Ageing (grant number 253).
